# Long-Term Consequences of High Titer Neutralizing Antibodies to Interferon-β in Multiple Sclerosis

**DOI:** 10.3389/fimmu.2020.583560

**Published:** 2020-10-15

**Authors:** Nicky Dunn, Anna Fogdell-Hahn, Jan Hillert, Tim Spelman

**Affiliations:** ^1^Department of Clinical Neuroscience, Karolinska Institutet, Stockholm, Sweden; ^2^Clinical Neuroimmunology, Center for Molecular Medicine, Karolinska Institutet, Stockholm, Sweden

**Keywords:** neutralizing antibodies, interferon-β, multiple sclerosis, immunogenicity, annual relapse rate

## Abstract

**Background:**

Neutralizing anti-drug antibodies (NAbs) to interferon beta (IFNβ) develop in up to 47% of multiple sclerosis (MS) treated patients inhibiting treatment effect of IFNβ. However, the long-term effect of NAbs remain unknown.

**Objective:**

To investigate the long-term consequences of high titer NAbs to IFNβ on disease activity and progression in MS patients.

**Methods:**

An observational study including data from all IFNβ treated relapsing remitting MS patients with sufficient NAb test results from the Swedish MS registry. Patients were classified into either confirmed ‘high titer’ or ‘persistent negative’ groups and analyzed for differences in disease activity and progression over time.

**Results:**

A total of 197 high-titer and 2907 persistent negative patients with 19969.6 follow up years of data were included. High titer NAbs were associated with a higher degree of disease activity at baseline. However, even when accounting for this, the presence of high titer NAbs were also associated with higher disease activity during IFNβ treatment. This persisted even after the next DMT start, suggesting that earlier high titers may partially reduce the effect of later treatments. No difference was found in confirmed disability progression.

**Conclusion:**

High titer NAbs to IFNβ are associated with higher disease activity, persisting even after IFNβ discontinuation or switch. These results support use of highly efficient treatment earlier in patients with active disease, to avoid these complications.

## Introduction

Neutralizing anti-drug antibodies (NAbs) can develop following treatment with recombinant human protein drugs (1). This includes interferon beta (IFNβ), used as a first line treatment in the management of multiple sclerosis (MS) since the 1990s (2). NAbs can diminish the biological activity of IFNβ, subsequently reducing clinical efficacy (3, 4).

The incidence of NAbs following treatment with IFNβ for multiple sclerosis can vary considerably with dose, frequency and product (5). Evidence from phase III clinical trials has observed a wide incidence range of NAbs; from 2.1–22.0% under intramuscular (IM) IFNβ-1a (6–10), 12.5–25.0% on subcutaneous (SC) IFNβ-1a (10–12), and 27.8–47.0% on IFNβ-1b (13–16).

Once high titer NAbs to IFNβ have been established, they may persist for many years and thus render patients unsuitable for IFNβ treatment, which thanks to the arrival of new MS disease modifying treatments (DMTs) has become a less critical problem with time (17–19). However, in 2010, van der Voort and colleagues reported that a group of NAb positive MS patients deteriorated more rapidly than expected over the following years, suggesting that the presence of IFNβ NAbs may in itself be a risk factor for MS progression by an unknown mechanism (20). They speculated that, by an effect on endogenous interferon pathways, NAbs may result in a more proinflammatory modification of the immune system leading to an increase in MS disease activity. Indeed, it was subsequently shown by (27), that IFNβ-treatment induced NAbs can actually neutralize endogenous IFNβ (21). However, the patients with persistent NAbs in their study did have indications of a more active disease prior to treatment start, although this was not significant in a small cohort of 71 patients. To our knowledge, no further studies have attempted to follow up on this observation in spite of its importance given that thousands of MS patients are rendered IFNβ NAb positive every year even today.

Whilst the availability and frequency of NAb testing has varied markedly across different European settings, such testing has been available in Sweden since 2003 for both screening and titration (22). As such, the Swedish MS registry (SMSreg) is uniquely placed to explore associations between antibody titer and later clinical outcomes. SMSreg is a longitudinal, observational MS outcomes database used across all Neurology departments in Sweden (23). The objectives of this study were to examine the association between confirmed high titer NAbs and annualized relapse rate (ARR), and time to first post-baseline relapse, confirmed disability progression and Expanded Disability Status Scale (EDSS) milestones, relative to patients with persistent negative titers.

## Materials and Methods

### Data Source

#### Swedish Multiple Sclerosis Registry

All data was sourced from the Swedish MS registry (SMSreg) on the 19th November 2016. SMSreg was established in 2000 to capture and collate clinical data on MS patients. Whilst neurologist participation in the registry operates on an opt-in basis, SMSreg is currently used in all neurology departments across Sweden, capturing approximately 80% of the prevalent Swedish MS population. A minimum dataset of mandatory variables is required for data upload and includes patient demography, diagnostic criteria, clinical visit details, treatment and relapse parameters (23). The data quality of in the SMSreg was assessed in a recent report (24).

#### Study Design and Patients

A retrospective observational cohort study was carried out using data from patients with relapsing remitting MS (RRMS) and aged 18 or older at baseline. To be included in the analysis, patients were required to have sufficient anti-IFNβ NAb test data recorded in the registry to permit comparison group classification. NAbs were measured as previously described with two different bioassays; the myxovirus resistance protein A (MxA) gene expression assay (MGA) and the commercially available iLite^TM^ anti-human IFNβ-1a bioassay (Biomonitor/Euro Diagnostica), which have been shown to be comparable (25). No minimum duration of pre-baseline DMT treatment or post-baseline follow up was required to be included. All patients who met the inclusion criteria at the time of data extraction were included. Follow-up duration was defined as the time from baseline titer test until the date of the last recorded clinical visit in the registry.

#### Comparison Groups and Definitions

**FIGURE 1:**
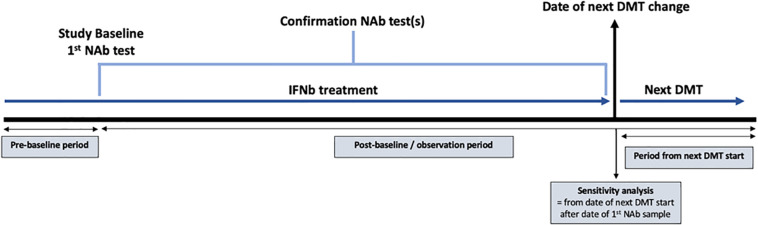
Diagram demonstrating the study design using data extracted from the Swedish Multiple Sclerosis registry. The study baseline was defined as the date of the first recorded high titer for the high titer group and the date of the first recorded negative titer for the persistent negative group. NAb = neutralizing antibody; IFNβ = interferon beta; DMT = disease modifying therapy.

Patients were classified into one of two comparison groups based on an initial assessment of 10,107 antibody titer tests from 5880 patients. The “confirmed high titer” group was defined as a neutralizing antibody titer ≥600 TRU/ml for a minimum of two consecutive tests. Whilst previous studies have considered a clinically relevant cut-off of ≥150 TRU/ml, we employed a stricter, higher cut-off to avoid transient positives (18). The median (IQR) time between the consecutive titers used to identify the “high-titer” group was 4.29 months (2.99, 8.77). The “persistent negative titer” group was defined as “never-positive” patients who recorded at least two negative screening test results or a titer under 10 TRU/ml in subsequent titration of neutralizing antibody tests with at least 24 months between the first and last test. Baseline for the confirmed high titer group was defined as the date of the first recorded high titer, whereas baseline for the persistent negative group defined as the date of the first recorded negative titer (Figure 1). Patients were censored at either the date of the event (depending upon the outcome being analyzed), the date of death, or the last observed assessment clinic visit.

#### Outcomes and Endpoints

The primary outcomes of this study were a comparison of ARR and time to first post-baseline relapse between the high-titer and persistent negative groups. Secondary, exploratory outcomes included 3-, 6-, and 12-months confirmed disability progression. Confirmed disability progression events were defined as ≥3-, ≥6-, or ≥12-months confirmed increases of ≥0.5 points for patients with a baseline EDSS score >5.5, ≥1.0 point for those with a baseline EDSS score between 1.0 and 5.5, inclusive, and ≥1.5 points for those with a baseline EDSS score of 0. EDSS scores recorded within 30 days after the onset of a relapse were excluded. Time to milestone EDSS 4 and 6 were also analyzed.

#### Statistical Analyses

Categorical variables were summarized using frequency and percentage and compared between titer groups via a chi-square test. Continuous variables were summarized using mean and standard deviation (SD) or standard error (SE), or median and inter-quartile range (IQR), and compared between titer groups using a *t*-test or Wilcoxon rank-sum test as appropriate. ARR was derived and compared for each treatment group using with confidence intervals (CI) calculated from the Poisson likelihood. Relapse count data were assessed for overdispersion. Generalized Estimating Equations defined on a Poisson family distribution were used to adjust ARR for age, sex, baseline EDSS, pre-baseline ARR and pre-baseline DMT exposure. Kaplan–Meier estimates were used to compare outcomes by titer group. A multivariate Cox proportional hazards regression model was used to analyze time to first relapse, confirmed disability progression and EDSS milestone adjusted for age, sex, baseline EDSS, pre-baseline ARR, and pre-baseline DMT exposure (count of pre-baseline DMTs and proportion of pre-baseline disease duration spent on DMT). Hazard proportionality was assessed via analysis of scaled Schoenfeld residuals. A sensitivity analysis was performed resetting the baseline for the analysis of the relapse outcomes from the original baseline (date of first observed high titer or first observed negative titer) to the date of the next DMT switch product following the first observed titer date. All modeling was based on a complete cases basis. For all analyses, *p* < 0.05 was considered significant. All analyses were conducted in Stata version 15 (StataCorp, College Station, TX, United States).

## Results

### Patients

A total of 197 confirmed high titer patients and 2907 persistent negative patients met the inclusion criteria and qualified for the analysis (Table 1). Patients in the confirmed high titer group were significantly older at baseline (mean 45.3 years, SD 11.6) relative to the persistent negative titer group (mean 40.9 years, SD 10.8) (*p* < 0.001). The high titer group reported fewer individual DMT starts prior to baseline (*p* = 0.005) although there was no difference in the proportion of pre-baseline disease duration spent on DMT therapy between the two titer groups (*p* = 0.127). The most frequent DMT at baseline reported by the high titer group was sub-cutaneous (SC) IFNβ-1a (65.0%) followed by IFNβ-1b (25.4%). By contrast, intra-muscular (IM) IFNβ-1a was the most frequent baseline DMT in the persistent negative titer group (52.4%) followed by SC IFNβ-1a (31.5%). Of those patients who switched therapy from baseline IFN during follow-up, the most frequent switch in the high titer group was to glatiramer acetate (50.8%), followed by natalizumab (8.6%), and an alternate IFN product (7.1%). By contrast, the most popular treatment switches in the persistent negative group was natalizumab (17.8%), alternate IFN (14.8%), rituximab (10.1%), and dimethyl fumarate (9.7%). Mean (SD) time between first and last recorded NAb tests was 3.7 years (1.5). The pre-IFNβ treatment ARR was significantly higher in the high titer group (0.27; 95% CI 0.24–0.29) relative to the persistent negative group (0.22; 95% CI 0.20–0.24). There was no significant difference in follow-up time between the high titer group (mean 6.72 years, SD 3.57) and the persistent negative titer group (mean 6.41 years, SD 3.30). Similarly, there was no difference in the count of post-baseline EDSS assessments between the high titer group (mean 8.04, SD 6.12) and the persistent negative titer group (mean 7.91, SD 5.19).

**TABLE 1:** Baseline characteristics by NAb titer group.

**Baseline characteristic**	**Category**	**Confirmed high titer (*n* = 197)**	**Persistent negative (*n* = 2907)**	***p*-value**
Sex – n (%)	Female	143 (72.6)	2108 (72.5)	0.982
	Male	54 (27.4)	799 (27.5)	
Age (years) – mean (SD)		45.3 (11.6)	40.9 (10.8)	< 0.001
Disease duration (years) – mean (SD)		10.4 (8.5)	9.0 (8.2)	0.004
DMT at baseline – n (%)	Avonex	19 (9.6)	1524 (52.4)	< 0.001
	Betaferon/Extavia	50 (25.4)	468 (16.1)	
	Rebif	128 (65.0)	915 (31.5)	
Count of pre-baseline DMTs excluding baseline DMT – mean (SD)		0.3 (0.8)	0.4 (0.8)	0.005
Proportion of pre-baseline disease duration on DMT – mean (SD)		0.8 (0.3)	0.8 (0.2)	0.127
Annualized Relapse Rate (pre-baseline) – ARR (95% CI)		0.27(0.25,0.29)	0.22(0.20,0.24)	< 0.001
Treatment switch product – n (%)	Alternate IFN	14 (7.1)	429 (14.8)	< 0.001
	Glatiramer	100 (50.8)	195 (6.7)	
	Natalizumab	17 (8.6)	518 (17.8)	
	Rituximab	4 (2.0)	293 (10.1)	
	DMF	4 (2.0)	281 (9.7)	
	Other	24 (12.2)	517 (17.8)	
	No switch	34 (17.3)	674 (23.2)	

*n, number; SD, standard deviation; CI, confidence interval; ARR, annual relapse rate; DMT, disease modifying therapy; IFN, interferon; DMF, dimethyl fumarate.*

### Annualized Relapse Rate (ARR)

During the full observation period, the high titer group (*n* = 197) had a significantly higher ARR (ARR 0.057 95% CI 0.045, 0.072) with a total of 76 relapses over a total of 1323.21 follow up years compared to the persistent negative group (*n* = 2907) which had 653 relapses over 18646.29 follow up years (ARR 0.035 95% CI 0.032, 0.038) (*p* < 0.0001). This difference remained significant after adjusting for baseline confounders. The confirmed high titer group was associated with a significantly higher post-baseline ARR relative to the negative titer group (Table 2) across the entire observation period. When disaggregated by baseline DMT this difference in ARR was further observed when the analysis was limited to baseline IFNβ-1b only (*p* < 0.0001), but not IM-IFNβ-1a (*p* = 0.2629) or SC-IFNβ-1a (*p* = 0.1108). ARR in the subset of the observation period prior to first post-baseline DMT switch was significantly higher in the high-titer group (ARR 0.074; 95% CI 0.061–0.088) compared with the negative titer group (ARR 0.045; 95% CI 0.041–0.048). Although adjusted for pre-baseline ARR, in the high titer group only, ARR was significantly higher (*p* < 0.01) in the observation period prior to first post-baseline DMT switch (ARR 0.074; 95% CI 0.061–0.088) compared to the subsequent on-treatment ARR (0.050; 95% CI 0.038–0.064).

**TABLE 2:** Comparison of ARR by titer group (full observation period).

			**Unadjusted**		**Adjusted**	
**Group**	**Total number relapses**	**Total follow-up years**	**ARR (95% CI)**	***p*-value**	ARR (95% CI)*	***p*-value**
High titer (*n* = 197)	76	1323.31	0.057 (0.045, 0.072)	<0.0001	0.055 (0.044, 0.070)	<0.0001
Persistent negative (*n* = 2907)	653	18646.29	0.035 (0.032, 0.038)		0.037 (0.034, 0.040)	

*Adjusted for age, sex, baseline EDSS, pre-baseline ARR, and pre-baseline DMT exposure. CI, confidence interval; ARR, annual relapse rate; EDSS, expanded disability status scale; DMT, disease modifying therapy.

### First Post-baseline Relapse

**FIGURE 2:**
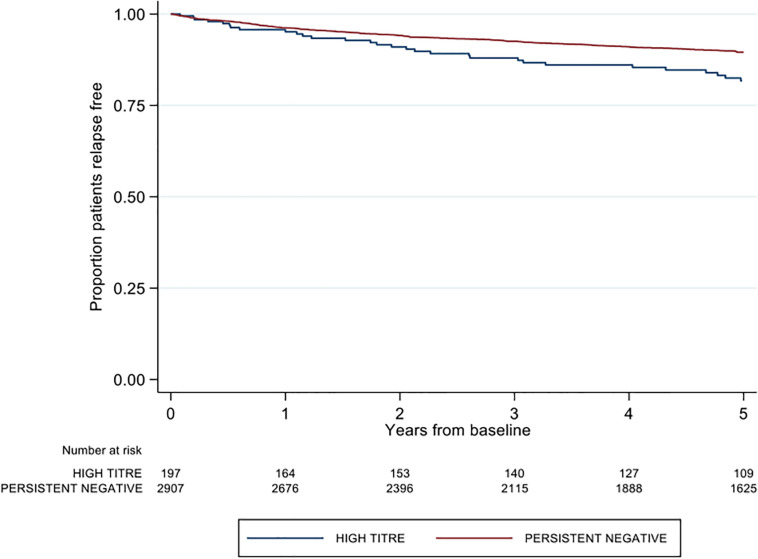
Kaplan-Meier curve demonstrating the rate of first post-baseline relapse by neutralizing antibody titre group over a five-year period.

A significantly higher proportion of patients in the high-titer group recorded a first post-baseline relapse relative to the persistent negative group (16.8 vs. 11.7%; *p* = 0.033). The majority of these relapses in the high-titer group occurred whilst on treatment with index IFNβ (27/33, 81.8%). The proportion of first relapses occurring on index IFNβ treatment was significantly less in the persistent negative titer group (191/339, 56.3%; *p* = 0.005). The high titer group was further associated with 1.48 times the rate of first post-baseline relapse relative to the negative titer group [Hazard ratio (HR) 1.48; 95% CI 1.03, 2.11; *p* = 0.033], adjusting for age, sex, pre-baseline ARR, proportion of disease duration on DMT and baseline EDSS (Figure 2).

### EDSS Outcomes

**FIGURE 3:**
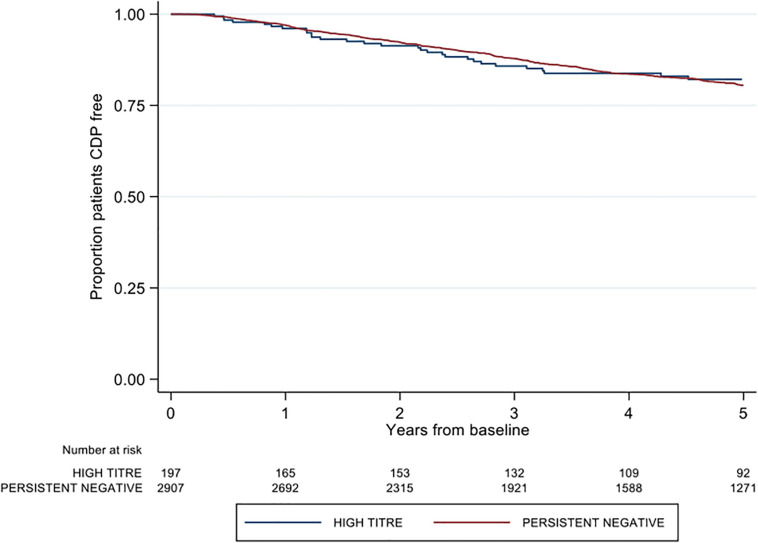
Kaplan-Meier curve demonstrating the rate of time to first post-baseline three month confirmed disability progression by neutralizing antibody titre group over a five-year period. CDP = confirmed disability progression.

There was no difference between high and negative NAb titer groups in time to first post-baseline 3-months confirmed disability progression (adjusted HR 0.84; 95% CI 0.60–1.18; *p* = 0.313) (Figure 3). Similarly, no group differences were observed in either 6-months CDP (aHR 0.86; 95% CI 0.61–1.29; *p* = 0.373; reference = negative titer) or 12-months CDP (aHR 0.88 95% CI 0.62–1.25; *p* = 0.474). The confirmed high titer group was significantly associated with 1.51 times the rate of milestone EDSS 6 on adjusted modeling (aHR 1.51; 95% CI 1.01–2.27; *p* = 0.046) (Table 2). There was no difference in time to EDSS 4 by titer group (aHR 1.26; 95% CI 0.86–1.85; *p* = 0.231).

### Sensitivity Analysis

As a sensitivity analysis, the baseline for the analysis of the relapse outcomes was reset from the original baseline (date of first observed high titer or first observed negative titer) to the date of the next DMT switch following the relevant first observed titer date. The high titer group was again associated with a significantly (*p* < 0.01) higher ARR (0.050; 95% CI 0.038–0.064) when compared against the persistent negative group (ARR 0.032; 95% CI 0.029–0.036). However, unlike the primary analysis there was no difference in time to first relapse between the two titer groups (HR 1.02; 95% CI 0.41–2.55; *p* = 0.960).

## Discussion

Patients in the high titer group were associated with both an increase in ARR and a shorter time to first relapse relative to the persistent negative titer group. This significantly higher ARR in the high titer group persisted even after the analysis baseline was reset to the date of the next DMT start, suggesting that earlier high titers may partially negate the effect of later treatment.

Alternatively, a high NAb titer at baseline may be related to higher disease activity at baseline, which in turn may mitigate the effects of subsequent treatment, and indeed this was the case with ARRs of 0.050 and 0.032 in the high NAb titer and NAb negative patient groups. However, the significantly higher rate of first relapse in the high-titer group was adjusted for both pre-baseline ARR and baseline EDSS, meaning the observed higher relapse rate in the high titer group was independent of both of these factors. Still, we can here confirm that the patients who develop high titer NAbs do have a more active disease from the beginning, as was indicated in the van Voort study (20).

Our observation from the primary analysis of a significantly higher ARR and a significantly reduced time to first relapse may be explained by a preferential loss of IFNβ effectiveness in the high titer group or, alternately, an increase in ARR among high titer patients hypothetically due to the neutralization of endogenously produced IFNβ by NAbs (21, 26, 27). However, we cannot determine from the primary analysis alone which of these effects, if any, may be at work here. To better help interpret these results, we shifted the study baseline from the date of first observed inclusion titer to the date of the next DMT start. This sensitivity analysis was designed to help determine whether the higher ARR and increased rate of first relapse in the high titer group observed in the primary analysis was localized to the pre-treatment observation period, or whether the increased relapse risk persisted after IFNβ therapy had been discontinued. Whilst the difference in ARR between the high titer and negative titer groups was smaller in the sensitivity analysis when compared to the primary comparison, the high titer group was still associated with a 50% increase in ARR relative to the negative group. Despite the difference in first relapse rates being no longer significant on the sensitivity analysis, this persistently higher ARR in the high titer group across both the primary analysis and sensitivity check suggests that high titer neutralizing antibodies may indeed be harmful. Alternatively, the more active disease seen in these patients might be a risk factor for developing NAbs, which subsequently would block the IFNβ therapy and thus contribute to the worsening of disease.

In contrast to the study from van der Voort et al., we found no evidence to suggest the differential effects observed in relapse-based outcomes extended to disability (20). No difference was observed in either confirmed disability progression or time to either EDSS 4 or 6 between the high titer and persistently negative neutralizing antibody groups. There are, however, differences between the two studies. First, our study was considerable larger high titer NAb positive patient cohort with 197 patients than the compared to the 17 patients available for comparison, allowing our survival analysis to be adjusted for several base-line confounders, including pre-treatment relapse rate, as well as for later treatment. In particular, our patients were likely to be much younger given the smaller risk of reaching EDSS 6. Second, the follow-up time in our study was longer (mean 6.4 years, maximum 12.8 years). In conclusion, regarding progression rates, the studies were not easily comparable. However, given that IFNβ treatment has less effect on disability progression (28), it is not unexpected that the NAbs similarly do not show any such effect.

Most importantly, our study, controlling for baseline confounders as well as for later treatment choices, confirms that development of persistent or high titer IFNβ NAbs are associated with an increased disease activity level. In addition, this risk is not sufficiently compensated for by future MS treatments. However, our observation of a significantly higher ARR in the high titer group when the analysis was restricted to baseline IFNβ only coupled with a significantly higher ARR in the high-titer group when the analysis was limited to the first post-baseline DMT period suggest this may in part be explained by a higher disease activity prior to the initiation of IFNβ treatment. All in all, this indicates that IFNβ treatment in MS, i.e. using a biosimilar to an endogenous protein component of immune regulation (27), may be harmful in the long-term if neutralizing antibodies are induced, in parallel to what has previously been reported for treatment with human recombinant erythropoietin (29, 30).

This study does have some limitations. Whilst the observed differences in clinical outcomes by titer group are independent of those confounder variables included in the multivariate model, we cannot claim these differences are adjusted for variation in other potential important correlates of outcome, most notably baseline MRI lesion count and distribution. The exclusion of MRI metrics from the primary analysis was due to a large proportion of patients not reporting an MRI at the study baseline. Similarly, our analysis is not controlled for variation in post-baseline confounders. A longitudinal model or marginal structural model (MSM) that permits adjustment for both baseline and post-baseline confounders may provide a potential future validation of our analysis.

In conclusion, whereas IFNβ is generally regarded as a safe treatment, our confirmation of an untoward effect of triggered NAbs indicate that patients with very active disease should not be put on less efficient first line treatments associated with risk for anti-drug antibodies. These patients would especially benefit from a highly efficient second line treatment earlier to avoid complications induced by the treatment.

## Data Availability Statement

The data analyzed in this study is subject to the following licenses/restrictions: the data cannot be made publically available due to privacy regulations. Under the General Data Protection Regulation, the Swedish law SFS 2018:218, the Swedish Data Protection Act, the Swedish Ethical Review Act, and the Public Access to Information and Secrecy Act, these types of sensitive data can only be made available, after legal review, for researchers who meet the criteria for access to this type of sensitive and confidential. Requests to access these datasets should be directed to TS (tim.spelman@ki.se).

## Ethics Statement

The studies involving human participants were reviewed and approved by Stockholm Ethical Committee. The patients/participants provided their written informed consent to participate in this study.

## Author Contributions

ND, AF-H, and JH contributed to conceptualization, investigation, writing, review, and editing of the manuscript. TS contributed to conceptualization, investigation, statistical analysis, writing, review, and editing of the manuscript. All authors contributed to the article and approved the submitted version.

## Conflict of Interest

AF-H has received speaker’s fees from Pfizer, Biogen, Merck Serono, and Sanofi Genzyme. She has received unrestricted research support from, Biogen Idec and Pfizer. JH has received honoraria for serving on advisory boards for Biogen, Sanofi Genzyme, and Novartis and speaker’s fees from Biogen, Novartis, Merck Serono, Bayer-Schering, Teva, and Sanofi Genzyme. He has served as P.I. for projects, or received unrestricted research support from, Biogen Idec, Merck Serono, Teva, Sanofi-Genzyme, and Bayer-Schering. His MS research is funded by the Swedish Research Council and the Swedish Brain foundation. TS has received compensation for serving on scientific advisory boards, honoraria for consultancy and funding for travel from Biogen Inc., speaker honoraria from Novartis. The remaining author declares that the research was conducted in the absence of any commercial or financial relationships that could be construed as a potential conflict of interest.
